# A Review of Consequences of Poverty on Economic Decision-Making: A Hypothesized Model of a Cognitive Mechanism

**DOI:** 10.3389/fpsyg.2017.01784

**Published:** 2017-10-11

**Authors:** Matúš Adamkovič, Marcel Martončik

**Affiliations:** Institute of Psychology, Faculty of Arts, University of Prešov, Prešov, Slovakia

**Keywords:** poverty, scarcity, poverty trap, cognitive load, executive functions, economic decision-making, time-discounting, risk preference

## Abstract

This review focuses on the issue of poverty affecting economic decision-making. By critically evaluating existing studies, the authors propose a structural model detailing the cognitive mechanism involved in how poverty negatively impacts economic decision-making, and explores evidence supporting the basis for the formation of this model. The suggested mechanism consists of a relationship between poverty and four other factors: (1) cognitive load (e.g., experiencing negative affect and stress); (2) executive functions (e.g., attention, working memory, and self-control); (3) intuition/deliberation in decision-making; and (4) economic decision-making (e.g., time-discounting and risk preference), with a final addition of financial literacy as a covariate. This paper focuses on shortfalls in published research, and delves further into the proposed model.

## Introduction

Poverty is a global socio-cultural phenomenon usually examined from an economic perspective. In behavioral studies, research on poverty largely focuses on the familial and social aspects of the background of people experiencing poverty, with the majority of research being carried out on children. Behavioral focuses of poverty research have included the psychological determinants of poverty, as well as the consequences of poverty on the mental health and cognitive functions of individuals (see Džuka et al., 2017). In a recent study, Haushofer and Fehr (2014) pointed out the existing need to direct attention toward the currently neglected issue of poverty perpetuation, which has generally been overlooked in favor of assessing the poverty from a solely economical (or even macroeconomical) perspective (see e.g., Semmler and Ofori, 2007; Naschold, 2012; McKay and Perge, 2013). Thus, it is clearly of importance to further examine the factors that may potentially help to unveil underlying reasons for poverty perpetuation. These factors include specific aspects of an individual’s perception of issues, personal experiences, behaviors, and individual abilities, which can either contribute to, or attenuate poverty. According to Mani et al. (2013), poverty perpetuation is likely the outcome of the interplay of various forms of non-productive behaviors such as inappropriate economic decision-making, or lack of own healthcare. These factors, in particular those related to economic decisions, are often labeled as causes of poverty. In this paper, we suggest that a circular relationship might exist between the causes and consequences of poverty, with the consequences of poverty (e.g., negative affect, stress, or impeded cognitive functions) simultaneously acting as poverty triggers, thus creating a poverty cycle also known as a poverty trap.

Based on the aforementioned research conducted by Mani et al. (2013), and Haushofer and Fehr (2014), it is possible to determine that examining the relationship between poverty and economic decision-making (as a consequence of poverty) is necessary to explain the underlying psychological aspects of poverty perpetuation. At the same time, efficient research on this issue should take care to pay heed to other variables not discussed here, which may have the potential to influence the poverty-economic decision-making relationship. Thus, the aim of this review is to propose a theoretical framework for the poverty-economic decision-making relationship, and to further explore economic decision-making as a consequence of poverty on four basic levels. Namely: (1) the effect of poverty on cognitive load experience (negative affect and stress); (2) the effect of poverty on executive functions (attention, working memory capacity, self-control capacity); (3) intuition/deliberation in decision-making; and (4) the effect of poverty on economic decision-making (time-discounting, and risk preferences related to reward/loss). Note that these levels are not mutually exclusive, but affect each other in different ways. Following this literature review, a proposed structural model integrating the aforementioned levels into one complex system will be laid out.

## Poverty Definition and Assessment

Our analysis of mainly psychological literature revealed that poverty is primarily regarded as an economical construct, and subsequently a psychological (or socio-behavioral) one. The exact operational (or conceptual) definition of poverty remains undecided upon, with most literature lacking a precise definition of the construct. As a result, the most currently relevant definitions of poverty are those proposed by global organizations. The United Nations (1995), for instance, defines general poverty as a complex construct of factors such as income insufficiency, lacking resources to ensure dignified living, experiences of hunger, aggravated health and poor healthcare, limited access to education, improper housing conditions, and social discrimination. The World Bank (in Haughton and Khandler, 2009) further defines poverty in a similar manner but goes on to delineate the psychological aspect of poverty by discussing matters of subjective well-being. However, these definitions remain rather ambiguous and open to questioning. For example, how might one define “dignified living”? What exactly might improper housing constitutes? Where might the line be drawn between the availability of food being accessible or limited? Poverty, therefore, appears to be a multidimensional construct which presents itself with various aspects that can be assessed both on an individualistic (subjective) level, as well as objectively, based on more general predefined criteria.

In practice, researchers tend to assess poverty according to various objective poverty lines (e.g., household incomes being lower than 60% of the country median or the income-to-needs ratio). However, poverty lines are not representative of whether or not individuals consider themselves to be poor (see Ravallion, 2016), a psychological aspect of poverty that should be heeded. Mani et al. (2013) found that a subjective experience of poverty is associated with deprived cognitive capacities to a greater extent than objective poverty indicators. Therefore, in order to assess poverty as a multidimensional construct (see: Smeeding, 2015), it is apparent that traditional assessments via economic indicators should be enriched by the inclusion of subjective evaluations of psychosocial measures of poverty such as subjective well-being poverty (Shams, 2015) or subjective social status (Diemer et al., 2013). Furthermore, as poverty is not a one-off state and is subject to temporal changes (Cooper et al., 2012; Dutta et al., 2012; Bresson and Duclos, 2014), it is necessary to measure individuals’ perceptions of the length of poverty duration, and the frequency of poverty reoccurrence across a lifespan.

## Poverty and Cognitive Load in the Form of Experiencing Negative Affect and Stress

Cognitive load refers to the presence of a burden on the cognitive system of an individual. An increase in cognitive load can occur when dealing with a problem and focusing attention on certain stimuli, thus leading to a reduced ability to attend to other stimuli (Paas and Van Merrienboer, 1994; Sweller et al., 1998). From the point of poverty research, an increase in cognitive load has been found to be associated with negative experiences related to long-term poverty (Shah et al., 2012). Moreover, a study by Haushofer and Fehr (2014) found that people living in poverty are more likely to experience cognitive load in the form of stress and negative affect, due to protracted exposures to adverse economic and social phenomena. Hence, negative affect and stress could be the bridging factor between poverty and its effect on economic decision-making (Haushofer and Fehr, 2014). From an economic context, cognitive load can arise from a person living in poverty having to deal with constant uncertainties in current and future economic situations. As coping with the resulting negative affect reduces one’s cognitive resources, this can lead to a deterioration of executive functions, thus causing an individual to become enmeshed in a cycle of focusing on poverty-related problems (see Shah et al., 2012).

Negative affect and stress are consequences of both persistent financial pressure and associated economic vulnerability (McLeod and Kessler, 1990) as well as social dimension of poverty. For instance, people living in poverty may lack financial and social resources to cope with acute and chronic problems. This can lead to individuals having to deal with negatively skewed affective perceptions of situations on top of the negative situations themselves (Haushofer and Fehr, 2014). Evidence from longitudinal studies (Lorant et al., 2003; Najman et al., 2010) further reveal a direct connection between poverty and depression. Similarly, Kim et al. (2013) found that growing up poor leads to increased negative emotional experiences in adulthood.

The relationship between poverty and stress can be evaluated on two levels: (1) short-term, where poverty diminishes one’s ability to respond to threatening and unpredictable events (i.e., economic aspects such as loss of work) and (2) long-term, where an individual deals with an allostatic load (i.e., constant thinking about the financial situation). In both cases, cortisol production, a biological indicator of stress, (Blair et al., 2011), has been found to increase after as little as 1 year of living in poor financial conditions (Butterworth et al., 2011). Haushofer and Fehr (2014) further list several examples of experimental situations (see Fernald and Gunnar, 2009; Baird et al., 2013), which provide evidence for a causal effect of poverty on stress via indicators such as subjective evaluations, or cortisol levels.

As has been mentioned, poverty is highly correlated with the experience of both negative affect and stress across short- and long-term situations. This is also associated with cognitive load, and potentially with ego-depletion. While emotional well-being or cognitive evaluation of situations are directly related to poverty, we argue that the resulting cognitive load can impede crucial executive processes, specifically attention, working memory, self-control, and decision-making.

## Poverty and Executive Functions

The majority of studies addressing poverty and cognitive/executive functions have traditionally been administered to children (e.g., Evans et al., 2005; Ayoub et al., 2009; Dickerson and Popli, 2016; Kaya et al., 2016), with only a few authors (Shah et al., 2012; Mani et al., 2013) focusing on adults. Based on existing studies, we have isolated 3 executive functions that may play a crucial role in the mechanism linking poverty and economic decision-making. Namely, (1) attention, (2) working memory and (3) self-control (self-regulation) capacity. In respect to the presented findings, these executive functions are associated with not only poverty but also with its consequences on cognitive load.

### Attention

Attention is the ability to select and focus on relevant information in the environment, whilst ignoring other information of lesser task-related importance (Kastner and Pinsk, 2004; Lui and Tannock, 2007). The effect of poverty on attention has been examined in a series of experiments conducted in both simulated (e.g., by inducing resource restriction and a sense of poverty in games; Shah et al., 2012; Mani et al., 2013), as well as in real-world environments (e.g., in pre- and post-harvest measures of cognitive functions of Indian farmers; Mani et al., 2013). As Shah et al. (2012) discovered, people deprived of resources less engaged in games, were fatigued, and took longer to make a decision, while also scoring worse on an attention test than controls. They argued, therefore, that the scarcity of any kind of resource can lead to an excessive degree of engagement with a task. This focusing of attention on certain problems (e.g., states of deprivation like hunger, or task-related time-pressures) can lead to attentional neglect of other stimuli. Specifically, in a difficult economic situation, this narrowing of attention may lead to problematic decision-making, such as the incautious borrowing of money (e.g., people living in poverty often make use of short-term high-interest loans), late bill payments, and even making heedless purchases.

One’s attention can also be impaired by cognitive load. For instance, Mani et al. (2013) found that (1) experiencing financial pressures can lead to higher exhibited stress levels, (2) cognitive functions (i.e., attention and intelligence) are significantly lower before (temporarily) resolving financial difficulties, (3) cognitive performance is negatively correlated with the severity of financial difficulties experienced, and (4) these results hold true even when factors such as physical exertion, anxiety, nutrition, or learning effects on test performance are controlled for. According to the authors, the mechanism of highly focused attentional capture caused by poverty is, therefore, the most significant factor in the reduction of cognitive performance. The authors also highlight the importance of distinguishing long-term poverty from short-term scarcity. While long-term poverty affects cognitive load due to the chronic experience of negative emotional states, scarcity is best qualified as an acute dearth of resources leading to a temporal increase in cognitive load by tempting one to immediately satisfy a need, while disregarding future costs (Shah et al., 2012).

Besides this, attention can be also influenced by stress. Despite the finding that stress does not fully explain the observable decline of cognitive functions, Mani et al. (2013) identify the mechanism of poverty with a broader concept of stress. The authors claim that aspects of scarcity become the key focus of individuals’ attention, leading to obsessive thoughts and an eventual reduction of mental resources. Meanwhile, Braunstein-Bercovitz (2003) argues that cognitive load also increases selective attention to stressors, amplifies stress levels, and is detrimental to the ability to diffuse attention to other relevant issues. It is plausible, that stress affects attention in different ways (in certain cases it can help to focus on relevant stimuli, see Chajut and Algom, 2003) relative to its attributes such as a concrete type of stressor or the duration of its exposure.

### Working Memory

Working memory is the ability ‘to hold information in mind and mentally work with, while this information is not accessible by a sensory apparatus at that moment’ (Diamond, 2013, p. 142). The majority of research on working memory and poverty has been conducted on children, revealing that living in poverty causes significantly worse working memory (Tine, 2014; Pavlakis et al., 2015; Rowe et al., 2016). From a biological perspective, this may be the result of reduced hippocampal development often associated with low socioeconomic status (Pavlakis et al., 2015). Engel de Abreu et al. (2014) further propose two psychological explanations. Mainly, poverty diminishes working memory due to insufficient cognitive stimulation. Moreover, the lower test scores of children in poverty compared to financially secure children may result from standardized tests being inappropriate to their social-cultural background. When ‘culture-fair’ tools (e.g., digit-span test) are applied, differences are often abolished.

A longitudinal study by Evans and Schamberg (2009) revealed that childhood poverty is correlated with decreased working memory in young adults, with stress (allostatic load) acting as a mediator of the relationship. The authors suggest a causal relationship in this case, as working memory was not found to be a significant mediator of the poverty-allostatic load relationship in an alternative model. Evans and Fuller-Rowell (2013) further confirmed that poverty and stress influence working memory, but argue that this effect is driven by self-regulation. Although short-term stress exposure (linked with task demands and duration) can facilitate working memory (Yuen et al., 2009), the effect does not apply to long-term exposure (e.g., poverty; Joëls et al., 2006).

The relationship between working memory and negative affect has been also examined. Brose et al. (2012) conclude that working memory is not a stable disposition, and fluctuates depending on negative affect (e.g., increased negative affect is related to diminished working memory performance), reduced control of attention, and motivation. The authors explain this with the allocation model (Ellis and Ashbrook, 1988), under which people experiencing negative affect end up focusing their attention on it, with subsequent attempts on self-regulation further limiting their mental capacities.

Despite the fact that ruminating on financial difficulties impairs performance, and requires intensive working memory involvement, it is possible that it may not necessarily impair cognitive functions related to proceduralized processes (Dang et al., 2015). In a recent study, Dang et al. (2016) showed that financial demands and consequent distractions diminish the cognitive functions of poor individuals. They argue that this impairment results from an overwhelmed working memory due to economic concerns. However, in certain conditions, these distractions can improve proceduralized processes such as learning (Markman et al., 2006). Dang et al. (2016) propose that these findings support the notion of learning through repetition and conditioning in poor people. Yet, it is unclear how effective this would be in real-world conditions of poverty (e.g., during economic decision-making). Without proper external control, it is possible that this process could easily facilitate inadequate economic behaviors instead.

### Self-control capacity

Diamond (2013) defines self-control as an individual’s ability to regulate attention, thoughts, behaviors, and emotions, by resisting temptations and impulsive behaviors (note: a broader concept is self-regulation; McCullough and Willoughby, 2009). Psychological theory offers several models of self-control, two of which we have selected as possible frameworks to explain the deterioration of self-control in relation to poverty. The Resource Model (Baumeister et al., 1994) describes self-control as an inner capacity-limited resource that can be exhausted when controlling one’s own behavior. Resisting one temptation, therefore, increases the chance of succumbing to a subsequent desire (Hofmann et al., 2012; Vohs, 2013). On the other hand, the Process Model (Inzlicht and Schmeichel, 2012) questions the existence of inner depletable resources. Instead, self-control is considered as a value-based decision-making process, with failures in self-control occurring due to shifts in motivational orientation, and attentional reorienting toward indications of potential rewards. Furthermore, Inzlicht and Berkman (2015) define the depletion of mental resources as a form of mental fatigue that prevents individuals from being able to motivate themselves to produce more effort.

Cognitive load can have a negative impact on self-control capacity. As poor individuals are constantly exposed to economic pressures (and must thus make extensive compromises in satisfying their desires, e.g., while shopping and during leisure time), their self-control capacity is correspondingly decreased. A persistent regulation of basic needs can thus lead to reduced self-control (Hofmann et al., 2012; Vohs, 2013). In addition to cognitive load, self-control is driven by attention and working memory. Baumeister et al. (1994) argue that directing attention away from oneself to the environment can lead to a loss of self-control. Mann and Ward (2007) claim that limited attentional resources cause individuals to focus on their acute needs and neglect more distal stimuli. Hence, such behavior does not correspond with optimal goals of self-regulation. Paradoxically, when urgent needs are associated with control and restriction, narrowed attention can lead to better self-control, with high working-memory capacity also enhancing self-regulation.

The depletion of mental resources for self-control can lead to impulsive and intuitive behaviors that eventually cumulate producing poor economic decisions, thus leading to a vicious cycle of poverty-inducing behaviors (Vohs, 2013). In contrast to previous research (see Heatherton and Wagner, 2011; Kurzban et al., 2013; Inzlicht et al., 2014), Dang et al. (2015) criticize the limited-resource model of self-control (Vohs, 2013), positing that self-regulation failures are due to motivation-based reasons instead of limited mental resources. Fundamentally, people become more sensitive to reward when financially deprived, thus stimulating a need for reward in other domains (e.g., making budget-exceeding purchases). At the same time, Tuk et al. (2015) conducted a meta-analysis of their own results and revealed that self-control in one domain may result in increased self-regulation in other potentially unrelated domains. Despite this strong evidence, however, they suggest that self-control may be dependent on the nature of the stimulus or task being dealt with. Thus, results from short-term interventions are likely not applicable to conditions of poverty, which may be chronic and/or episodic. Further investigation on the effect of poverty and its direct consequences in the form of negative affect and stress, together with the effect of attention and working memory on the self-control capacity would, therefore, be beneficial to further explore this topic.

On the whole, we believe that the Resource Model is more appropriate to explaining improper economic behaviors in the context of poverty. This is due to the fact that (1) it posits that mental capacity can be exhausted; this is applicable to circumstances of poverty, which impair working memory and attention; (2) we consider poverty perpetuation to be the result of a series of events rather than a failure in one’s motivation to expand more effort. Nonetheless, according to the latest evidence (Lindner et al., 2017), both self-control models explain the reduction of performance in subsequent tasks equally well.

To summarize, research has shown that poverty impacts executive functions directly, and indirectly via cognitive load in the form of negative affect and stress. In order to accomplish the goals of this review, three executive functions (self-control, attention, and working memory) gleaned from scientific literature were selected for further examination. Based on existing studies, we suggest that these executive functions have effect on economic decision-making. While the nature of their relationship remains unclear, we propose several alternative mechanisms of these relationships: (1) self-control depends on attention and working memory; (2) attention and working memory are dependent on self-control, (3) self-control, working memory and attention covary on the same hypothesized level, or (4) the functions reciprocally affect each other, with self-control being the most closely linked to economic decision-making.

## Intuition/Deliberation As A Determinant of Economic Decision-Making

Another process that influences economic decision-making is an individual’s intuitive/deliberative decision-making style. This intuition/deliberation dichotomy represents two distinct systems of thinking based on Dual process theory (the theory of two disparate reasoning processes; Evans, 2003; Kahneman, 2003, 2011). Kahneman (2011) defines the intuitive system of thinking as fast, implicit and heuristic-based, while the deliberative system is slow, rational and logical.

However, the capacity to make rational decisions does not translate to their actually being carried out (Starcke and Brand, 2012; see also Epstein et al., 1996; Kahneman, 2003). For instance, exposure to stress causes one to rely on simpler, more primitive automatic decision-making preferences (Porcelli and Delgado, 2009). The use of heuristics may be beneficial, as they are fast, accessible, and require less effort and resources (Hafenbrädl et al., 2016). In particular, the framing effect heuristic, or the manner in which one’s decisions are affected by the way that alternative choices are presented, may be more influential when under stress (Starcke and Brand, 2012). Evidently, stress impairs deliberative processes, reducing one’s ability to evaluate pros and cons of alternative choices (Simonovic et al., 2016). Moreover, Cui et al. (2015) propose that emotional experiences and stress pose high demands on working memory, resulting in poor decision-making abilities. The effect of stress on the use of intuition/deliberation in decision-making is presented by Yu (2016) in a stress-induced deliberation-to-intuition (SIDI) model. A meta-analysis by Fields et al. (2014) supports the existence of moderate to strong relationships between stress and impulsive decision-making. Furthermore, a study by Masicampo and Baumeister (2008) provides evidence that people under stress tend to opt for automatic instead of controlled processes when making decisions.

The relationship between self-control (self-regulation) and decision-making was examined by Pocheptsova et al. (2009). The authors suggest that self-control and decision-making share a mental capacity, and provide evidence that participants utilize simpler and more intuitive decision-making strategies following self-regulation (which depletes mental resources). Similarities also exist between the two systems of self-control described by De Ridder et al. (2012), and the theory of two processes of reasoning during decision-making. According to De Ridder et al. (2012, p. 78), self-control (self-regulation) follows either (1) a ‘cool’ pragmatic system or (2) a ‘hot’ feeling system. The pragmatic system determines one’s behavior based on rational evaluation (“do it if it makes sense”) and is associated with high self-control and low impulsive decision-making. The ‘Hot’ system, however, is regulated by a feeling principle (“do it if it feels good”) and is linked with low self-control and higher impulsivity.

According to research by Evans (2010), deliberative (analytical) processes depend on working memory (which is tightly linked to attention and executive functions), while intuitive processes are independent of it. Likewise, Travers et al. (2016) propose that deliberative reasoning depends on working memory capacity and self-control, and is influenced by mathematical abilities and dispositional factors. Contrarily, intuitive reasoning is independent of these factors.

When making a decision, people living in poverty must take into consideration a broad spectrum of compromises related to the economic and social aspects of poverty. These decisions tend to be intuitive, impulsive and poorly thought out. As resisting temptation deprives one of self-control resources (Vohs, 2013), this causes a chain reaction of future inappropriate decisions (see Shah et al., 2012). Therefore, we can conclude that intuition/deliberation in decision-making has the potential to mediate the relationship between executive functions induced by poverty and economic decision-making.

## Poverty and Economic Decision-Making

Behavioral economics offer several alternative forms of economic decision-making assessments, extending to the perspective of psychological research. For the purpose of this review, we focus on three pertinent aspects: (1) time-discounting; (2) risk-taking for potential reward; and (3) risk-taking with potential loss. In order to best assess the economic dimension of these aspects, it appears necessary to take individuals’ financial literacy into consideration. Controlling for financial literacy allows us to determine if economic preferences are either (1) a consequence of a cognitive mechanism of the effect of poverty, or (2) a consequence of financial literacy and the ability to utilize mathematical abilities during fundamental economic events.

### Time-Discounting

Time-discounting (similar terms: intertemporal choice, temporal discounting, delay discounting, delay of gratification) refers to decision-making which involves compromises between costs and benefits occurring at different times (Frederick et al., 2002), and is also a demonstration of self-control and will (Shamosh and Gray, 2008). According to Mishra and Lalumière (2016, p. 769), increased time-discounting indicates a ‘preference for smaller immediate rewards over larger, distal rewards.’

A study by Brown et al. (2015) describes the determinants of time-discounting. The authors found that people prefer larger delayed rewards if they have a higher income, are not liquidity constrained and are healthier/have longer life expectancy. Similarly, Carvalho et al. (2016b) found that a sense of financial stability in related to a willingness to wait for a higher reward, and to improved self-control. Furthermore, Liu et al. (2012) note that the choice of reward is influenced by psychological dimensions of poverty rather than by the objective socioeconomic status. They claim that the preference of a smaller immediate reward is due scarcity of resources faced by poor people, who are often at risk and have reduced self-control and display impulsive behavior. The authors hypothesize that immediate rewards are chosen to level their playing field with richer people, if even for an instance (see also Hoel et al., 2016).

Research on cognitive load and time-discounting yields fairly consistent results. Experiencing sadness (or negative affect) is associated with the preference of immediate, lower rewards (Lerner et al., 2013; Liu et al., 2013), whereas positive emotional states lead to the choice of higher, delayed rewards (Ifcher and Zarghamee, 2011; Liu et al., 2013). Neutral affect has no effect on the preference of the type of reward (Liu et al., 2013). Initially, stress was found to not have any effect on time-discounting. For example, Haushofer et al. (2013) induced a stress event in a laboratory setting, and observed no effect of stress on intertemporal choice. However, other studies (Cornelisse et al., 2013; Moreno, 2015) have since confirmed the impact of stress on a tendency to choose smaller and earlier rewards. Haushofer and Fehr (2014) explain this by postulating that: (1) stress leads to the favoring of habitual behaviors, and (2) earlier rewards come with higher satisfaction levels than delayed ones.

Research on time-discounting and working memory, however, has yet to reach a consensus. Shamosh et al. (2008) and Basile and Toplak (2015) report a positive correlation between time-discounting and working memory, with a decreased willingness to wait for a larger reward being related to diminished working memory. Contrarily, no such significant relationship was found by Steinberg et al. (2009). Similar issues can also be found with self-control. Waegeman et al. (2014) argue that a preference for larger, delayed reward is related to higher self-control. This is further supported by the findings in Basile and Toplak’s (2015) study which assessed the correlation between time-discounting and the ability to consider future consequences of decisions (a construct similar to the concept of self-control). Conversely, Carvalho et al. (2016a) state that despite scarce resources leading to inadequate economic behaviors, the expenditures of people shortly before and after payday do not differ. This, therefore, indicates that apparent self-control related differences are possibly due to liquidity constraints, rather than low self-control. Furthermore, according to Kidd et al. (2013), the willingness to wait for a larger reward depends on the perceived stability of one’s environment, instead of solely on self-control. Additionally, Michaelson et al., 2013) found that people tend to wait for a reward when others in the environment appear to be trustworthy. In other words, when a person believes the social context of a situation to be reliable, the likelihood of delayed gratification increases.

Studies on the relationship between deliberation or impulsivity in decision-making and time-discounting produce more consistent results. According to Frederick (2005), people who display higher deliberative reasoning in decision-making are more patient and thus prefer a higher reward, with this phenomenon being even stronger among women. However, the overall correlation was lower when the reward was only accessible after a long wait period (e.g., 10 years). Higher levels of deliberative reasoning in decision-making also predict better results in cognitive tasks, and reduce heuristic use and cognitive biases, while being linked to a preference for larger, delayed rewards (Travers et al., 2016). In line with this, Stanovich (2010) found that individuals’ intuitive/deliberative style of thinking predicts their performance in decision-making tasks (including reward preference) independent of other cognitive skills. Mishra and Lalumière (2016) postulate that the choice of smaller, earlier reward is correlated with the inability to control impulses (e.g., increased impulsivity, lower self-control, attraction to risky financial investments or vulnerability to gambling risks), with people living in poverty showing greater sensitivity to such behavior. And as noted by Wittmann and Paulus (2009), impulsive economic decisions are not generally sustainable in the long run.

We thus conclude that prior research provides evidence supporting the existence of a mechanism by which poverty induces cognitive load, impedes executive functions, and hence affects time-discounting. It appears evident that poor people favor immediate and smaller rewards that provide short-term satisfaction but are not economically beneficial from the long-term perspective. Since time-discounting is not the only aspect of economic decision-making, it is important to focus on risk preference as another factor.

### Risk Preference in Economic Decision-Making

Living in poverty is associated with an increased prevalence of risky behavior and potential negative consequences in areas such as health care (Shankar et al., 2010), sexual behaviors (McBride Murry et al., 2011), criminality (Hay et al., 2006), substance abuse (Datta et al., 2006; Mulia et al., 2008), and gambling (van der Maas, 2016). However, does this tendency apply to economic decision-making? Are people living in poverty more eager to accept the certainty of a smaller reward or might they willingly take the risk of losing a potential bigger reward? Finally, does a similar mechanism also work in the case of financial loss?

Andersen et al. (2008) claim that, in general, people naturally tend to avoid economic risk. Haushofer and Fehr (2014) further argue that the tendency to avoid risks related to financial rewards is even more pronounced in people influenced by poverty, as reward certainty can attenuate acute liquidity constraints. This decrease susceptibility to risk regardless of intrinsic risk preference. Experiments on people living in poverty conducted by Carvalho et al. (2016b) show that participants with bank accounts savings engaged in lottery risks (with potential financial rewards) more often. According to the authors, people with savings recognize the benefits of accumulating more money for future use. Finally, Carvalho et al. (2016a) did not find differences in reward related risk-taking between the groups of poor individuals before and after payday. They thus hypothesize that long-term (rather than short-term) financial stability may increase people’s willingness to take economic risks.

From the perspective of cognitive load, one can assume that exposure to naturally occurring negative emotions (fear and anxiety) might increase risk aversion in the case of reward (Heilman et al., 2010). Findings of the effect of stress on risk-taking are more ambiguous. Shah et al. (2012) claim that stress can lead to riskier economic decision-making. Similarly, Starcke and Brand (2012) state that stress (both experimentally induced and chronic) promotes risk-seeking preference for both reward and loss. However, other studies show that acute stress enhances conservative decisions when facing a potential reward (Porcelli and Delgado, 2009; Moreno, 2015), but also increases the chance of risk-taking in the case of potential loss (Porcelli and Delgado, 2009). Contrarily, Moreno (2015) found chronic stress to be virtually uncorrelated with risk preference in economic decision-making, while Kandasamy et al. (2014) found that induced chronic stress does contribute to risk-aversion.

A willingness to take risks is also linked with intuitive/deliberative style of thinking. Frederick (2005) found that individuals with more deliberative reasoning (higher cognitive reflection), were more inclined to risk-taking, especially when the potential reward was high. This was found to be more pronounced in men, with additional gender differences found – women with high scores in deliberative thinking were as prone to risk-taking as men with low scores in deliberative thinking. In the case of loss, individuals exhibiting deliberative thinking style were more willing to withstand a smaller loss compared to risking a larger loss. In contrast, people with a tendency for intuitive thinking were more inclined to take risks in case of a potential loss rather than a reward. Such behavior corresponds with the Prospect Theory (people are more sensitive to a loss than to a reward, risking more to avoid it; Kahneman and Tversky, 1979). This indicates that people do not consider risks in isolation, but also look at the profits (Cueva et al., 2016). Thus, people living in poverty prioritize ‘here and now’ rewards while simultaneously trying to avoid potential loss despite the fact that this can backfire, leading to even more negative consequences. This assumption can be explained in light of the influence of stress. Mather and Lighthall (2012) argue that acute stress initiates behaviors that have been rewarded in the past. However, when under stress, individuals’ perceptions of past negative experiences may be biased toward more positive evaluations. The willingness to take risks also differs across genders, with men being riskier (Mather and Lighthall, 2012; Cueva et al., 2016).

Therefore, we can conclude that the interacting mechanism of poverty, cognitive load and intuitive decision-making can lead to a tendency of the poor to risk less for a potential gain and simultaneously to risk more in case of a potential loss. According to Haushofer and Fehr (2014), risk aversion to rewards in poor people is separated from their intrinsic preference for risk-taking. Thus, the poor prefer guaranteed profits that help them to decrease liquidity constraints and compensate for frequently occurring negative events. In order to avoid financial loss, however, the poor may prefer to take risks. This has been explained by Mather and Lighthall (2012), who suggest that people under stress tend to avoid negative experiences and seek to prevent further negative consequences. Besides these factors, economic decision-making is also linked with financial literacy.

### Financial Literacy

Financial literacy is defined as an ‘ability to process economic information and make informed decisions about financial planning, wealth accumulation, debt, and pensions’ (Lusardi and Mitchell, 2013, p. 6). Moreover, it consists of a combination of apprehension, abilities, attitudes and behavior associated with economic aspects of life (OECD INFE, 2011).

Despite the fact that financial literacy directly affects economic decisions (Lusardi, 2011) and results in individuals with higher financial literacy being in better financial situations (Meier and Sprenger, 2013), education in this field is often neglected. Lusardi (2011) argues that illiteracy or ignorance toward basic financial concepts leads to incautious borrowing, or poor investments (e.g., in purchasing securities). Generally, people are unable to make simple economic calculations, lack knowledge about interest, cannot distinguish between a real and nominal product value, are not familiar with options of risk allocation, and have even less knowledge about more complex concepts. Lusardi and Mitchell (2011) and French and McKillop (2016) studied the financial management abilities and numerical skills of people in debt living in socially disadvantaged environments. This led to the discovery that wealth inequality is largely caused by deficiencies in financial management. However, mathematical abilities only had a marginal effect on financial situations. It was therefore concluded that better financial management abilities (as a component of financial literacy) result in a behavioral, rather than cognitive, benefit in helping individuals to cultivate a habit of borrowing less and avoiding high interest rates, thus decreasing overall debt.

The effect of financial literacy on economic decision-making has been described in several studies. Gathergood (2012) found that excessive financial demands and the inability to repay debts are correlated with impairments in self-control and, crucially, financial literacy. Moreover, Meier and Sprenger (2013) outlined a relationship between time-discounting and financial literacy, demonstrating that individuals with higher financial literacy prefer larger, delayed rewards.

In summary, poverty and economic decision-making are closely linked, with studies showing that poverty and cognitive load have an impact on economic decision making. Additionally, economic decision-making is associated with self-control and intuitive/deliberative style of thinking. In general, the evidence supports the notions that (1) individuals living in poverty are inclined toward smaller, earlier rewards due to higher cognitive load, lower self-control (higher impulsivity), and a tendency to utilize intuitive decision-making processes; (2) these characteristics are associated with a reluctance to take risks for a reward; (3) the same characteristics are related to a willingness to take risks associated with losses; (4) conversely, people who favor larger, delayed rewards are more willing to take risks associated with rewards and are more cautious in regards to potential loss; they also have higher self-control and/or more deliberative thinking style. These findings allow us to propose a complex conceptual model, which reflects the consequences of poverty on economic decision-making via a cognitive mechanism that rationalizes these relationships.

## The Proposal of Two Models Integrating Poverty, Cognitive Load, Executive Functions, Intuitive/Deliberative Style of Thinking and Economic Decision-Making

**FIGURE 1:**
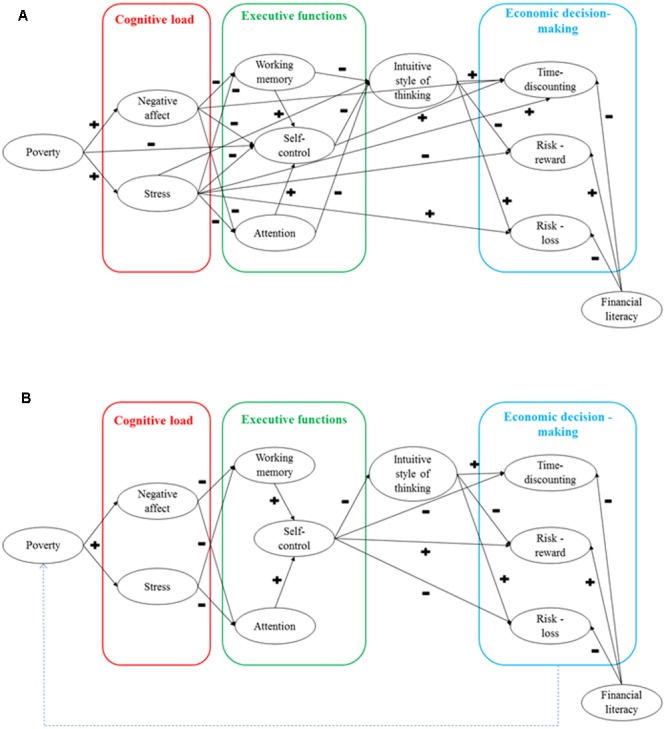
Two models of how poverty affects economic decision-making via cognitive mechanism. **(A)** The model integrating all the presented findings. **(B)** The proposed structural model of a cognitive mechanism of poverty perpetuation. + and - signs indicate the direction of the effect. E.g., the higher the self-control, the lower the tendency of intuitive style of thinking.

Based on the reviewed literature, we propose two models of cognitive factors that contribute to the perpetuation of the poverty cycle by negatively affecting people’s ability to make sound economic decisions. The first model consolidates all the presented prior findings (see **Figure 1A**). Since this model is too complex, we propose our own simplified version, which lays out the most probable causality of relationships in a comprehensive and parsimonious manner (**Figure 1B**). This model details a mechanism explaining difficulties faced in attempting to break out of the cycle. However, currently available empirical evidence is inconsistent, and the relationship between some variables remains to be assessed. Therefore, it remains necessary to build partial models and to examine their validity and parameters.

The mechanism of the predicted model (**Figure 1B**) can be explained as follows. Living in poverty, as defined by objective (e.g., person’s income, household income, wealth), and subjective indicators (e.g., subjective assessment of economic well-being and social status), is causally related to persistent, repeated and more prevalent states of negative affect and stress. In other words, living in poverty or having limited resources creates a heavy cognitive load in the form of negative psychological states such as shame, guilt, sadness, misfortune, fear, hostility toward others, uncertainty, worries, and distress. This mental pressure severely limits working memory capacity, and focuses attention on situations and needs that cannot be met because of poverty. This prevents these executive functions from being used on other problems, and causes these functions to seem deteriorated. Focusing on the emerging issues related to poverty, a person is constantly forced to choose which needs will and will not be satisfied, thus, leading to self-control being affected by the need to make compromises and resist temptations. This can result from a depletion of mental resources and/or the need to shift one’s attention and motivation from one task to another, thereby increasing vulnerability to impulsive behaviors for instant gratification. When a person is exhausted, deliberative processes are often neglected in favor of intuitive ones. This intuitive thinking style may be more beneficial in the short run, as it is automatic, based on heuristics, and requires little mental effort for decision making. Apart from conserving mental processes, intuitive thinking might also afford a hedonistic experience, as it provides an instant reward regardless of the potential consequences. The economic decision-making effect of intuitive thinking can also be seen in time-discounting tasks, compelling an individual to select a smaller immediate reward over a larger delayed larger one. This pattern of behavior could be explained in light of the fact that poverty and the cognitive mechanisms that result from it, encourage individuals to satisfy their emerging needs whenever possible. In risk preference tasks, the proposed mechanism can also result in cautious behaviors in regards to potential gain, and increased risky behaviors to moderate potential loss. This willingness to engage in risks when a potential losses are greater could be due to the fact that individuals in a period of poverty may already be facing significant issues, leading to any slight chance of loss being perceived as being disproportionately severe, and the possibility of no loss at all being seen as subjectively more beneficial. Altogether, these behaviors demonstrate the difference between psychological and economical rationality in decision-making. From an economic perspective, it is clearly more beneficial to wait for a larger reward. However, in the case of poverty, a long-run economic advantage may often be neglected in favor of satisfying present urgent needs which may otherwise be difficult to meet. From an evolutionary perspective, this pattern of behavior is justifiable, as primal motivations dictate that fundamental acute problems have to be met before long-term actions can be decided upon. However, this does not translate well to poverty alleviation, which requires the making of economic decisions that are focused not only on the “here and now,” but also take into consideration future consequences of decisions. As both time-discounting and risk preference have strong economic foundations, they are likely influenced by financial literacy. Therefore, it is necessary to control for its effect on economic decision-making. Although the proposed model focuses on cognitive mechanisms that arise from poverty, the outcomes of economic decision-making also create a feedback loop on future financial situations, leading to a cycle of poverty perpetuation.

## Potential Limits of the Presented Model

Validating the suggested model may run into several limitations, however, with the first being related to the methods of assessing poverty. Considering that poverty can be examined in our model from various perspectives, e.g., (1) through a focus on its subjective experience; (2) through a focus on its objective indicators; (3) by multidimensional approach combining psychological and economic indicators (likely the best solution) or (4) by categorically dividing people across a poor/not poor threshold (e.g., based on household income), with poverty becoming a moderator. However, such dichotomization omits subjective indicators crucial for the function of the overall model and could potentially reduce the proposed mechanism as early as the relationship between poverty and cognitive load.

One other issue is that of causality. Despite the fact that experimental evidence for the causes of poverty exists, the majority of existing research is based on statistical correlations between poverty and the aforementioned factors. Current research therefore only provides a tenuous hypothetical account for the causalities underlying poverty. To ensure that the model (and emerging partial models) are valid, the proposed mechanism is based on a factor of causality that we believe is the more likely, based on the presented literature. Namely, our proposed model suggests that poverty is the causal factor for the development of cognitive mechanisms underlying poor economic decision-making. However, an alternative hypothesis treats poverty as a consequence instead of the cause of different poverty-related processes, including those discussed in the text. For instance, cognitive abilities can affect economic outcomes, with higher intelligence being related to better jobs and higher incomes (Gottfredson, 1997). As we are aware that any kind of model merely attempts to simplify and reflect real-life events taken from a complex reality, we believe that the alternative models of the whole mechanism and of its parts should be tested as well.

Moreover, the results of previous studies are not always consistent. The most pertinent issue appears to be that of the relationship between cognitive load and executive functions. Currently, there is a lack of strong evidence as to how attention, working memory and self-control affect each other in situations of stress or negative affect (note: the description of the whole model includes the most plausible alternative). In order to clarify this system of relationships, it is thus necessary to test different partial models. We propose testing four alternatives: (1) working memory and attention as mediators of the relationship between cognitive load (negative affective and stress) and self-control; (2) self-control as a mediator between cognitive load and working memory with attention; (3) attention, working memory and self-control are at the same level, mutually covaried, and depend on cognitive load; and (4) attention, working memory and self-control affect each other reciprocally (creating non-recursive relationships) and depend on cognitive load.

Taking into account the various aspects of economic decision-making, time-discounting and risk preference/aversion to reward or loss into consideration, can also be problematic. Choi et al. (2014) provide evidence that individuals with limited mental resource capacity make inconsistent decisions. However, this can be overcome with the use of appropriate measurement tools (see Falk et al., 2016). Successfully distinguishing between economic and psychological rationality in financial decision-making (see Simon, 1993) can also be another potential issue. By its nature, economic decision-making is based on the principle of achieving maximum profit while minimizing potential loss. Hence, the result of such decisions can be evaluated mathematically. Conversely, psychological aspects of decision-making cover a wider range of decision-based contexts. For example, it is economically more rational to select the larger but delayed reward when given a choice between a reward of 100 now or 200 in a month. Nonetheless, such a conclusion simplifies and neglects the psychosocial aspects of decision making. For instance, one might choose an immediate reward to satisfy an urgent need such as purchasing food. Reducing economic decision-making to the simplified pursuit of economic advantage while neglecting more complex perspectives, therefore, becomes inadequate for the purposes of explaining and interpreting observed relationships or potential causalities.

In the presented overall model as well as in the partial ones, other variables may play an important role – e.g., gender, duration of poverty spell (eventually the number of poverty cycles in the lifetime) or type of social unit focused on when assessing poverty (individual vs. family). It would, therefore, be of benefit to at least control for participants’ social backgrounds, even in the case of research on the level of the individual.

Although we aimed to present a highly complex model, it was impractical to assess and analyze all possible variables that might plausibly interfere with the presented mechanism. Therefore, the model does not include variables such as: (1) intelligence, which is associated with time-discounting (Shamosh and Gray, 2008; Steinberg et al., 2009) and economic decision-making in general (see Rustichini, 2015); (2) time perception, which is also related to time-discounting (Soman et al., 2005); (3) the ego-depletion effect, which differs from cognitive load (Maranges et al., 2017), and might only occur under specific circumstances; and also a broader concept of fatigue; (4) motivation influencing self-control (Dang et al., 2015); (5) macroeconomic and political expectations toward future (Brown et al., 2015), or (6) metacognitive abilities, for example, feeling of rightness of judgment, which may determine the tendency of intuitive/deliberative decision-making (Thompson et al., 2011). Implementation of these variables into models can, therefore, be explored in future research.

In respect to existing research, this model is applicable in the context of poverty, as well as for evaluating the broader issue of (socio)economic status. However, the model might not be relevant in specific regions or countries affected by extreme poverty.

## Conclusion

Poverty is a serious long-term, pervasive issue in society. Although research on poverty has primarily been conducted from the perspective of economic science, present attention has shifted to more psychological aspects, focusing on the causes and consequences of poverty. As stated by Haushofer and Fehr (2014), the examination of such aspects can be a key to unraveling the processes leading to poverty persistence. Till recently, research has only been carried out in partial studies with distinct goals, inadvertently overlooking the role of relationships between different aspects across a broader framework. We thus propose a comprehensive holistic mechanism, detailing the manner in which poverty affects economic decision-making via cognitive load, executive functions and intuitive/deliberative style of thinking. Testing this model can thus be an initial step in attempting to explain the self-perpetuating nature of the poverty cycle.

This review contributes to current literature by bridging the gaps of missing connections between various aspects, which taken together as a system, can be used to examine the economic decision-making style of an individual. At the same time, further analysis of specific relations between poverty, cognitive load, executive functions, and economic decision-making can contribute to an understanding of events related to individuals and poverty. In a broader context, a greater understanding of the workings of specific poverty-related mechanisms also carries with it the potential to better craft and improve intervention programs focused on poverty alleviation.

## Author Contributions

All authors listed have made a substantial, direct and intellectual contribution to the work, and approved it for publication.

## Conflict of Interest Statement

The authors declare that the research was conducted in the absence of any commercial or financial relationships that could be construed as a potential conflict of interest.
